# Recharge net metering (ReNeM) is a novel, cost-effective management strategy to incentivize groundwater recharge

**DOI:** 10.1038/s44221-023-00141-1

**Published:** 2023-10-18

**Authors:** Molly Bruce, Luke Sherman, Ellen Bruno, Andrew T. Fisher, Michael Kiparsky

**Affiliations:** 1 Center for Law, Energy and the Environment, Berkeley School of Law, University of California, Berkeley, CA USA; 2 Global Policy Lab, University of California, Berkeley, CA USA; 3 Department of Agriculture and Resource Economics, University of California, Berkeley, CA USA; 4 Department of Earth and Planetary Sciences, University of California, Santa Cruz, CA USA

**Keywords:** Economics, Environmental studies

## Abstract

Managed aquifer recharge, which uses available water to augment groundwater resources, holds promise as a strategy to reduce chronic groundwater overdraft. However, water management agencies often confront hurdles when implementing managed aquifer recharge. Favourable sites for recharging water are often located on private land, and common-pool resource conflicts frequently disincentivize voluntary private participation. We introduce recharge net metering (ReNeM), a conceptually novel, market-based mechanism to help overcome these barriers and achieve multiple extractive and non-extractive benefits from improved groundwater management. ReNeM enables an agency to incentivize practices that enhance infiltration and groundwater recharge. Here we formalize the basis for incentivizing recharge and conduct a multi-party cost–benefit analysis of an operating ReNeM programme in California’s Pajaro Valley. Calculations show that water supply from ReNeM can be achieved at a lower cost than many viable alternatives and can produce multiple benefits for collaborating entities and stakeholders.

## Abstract (web summary)

Recharge net metering is an adaptable, performance-based incentive for groundwater recharge. It costs less than most methods, supports distributed recharge and imparts substantial benefits to both groundwater basins and programme participants.

## Main

Unsustainable groundwater management is a growing global concern with unsettling environmental, economic and social implications^1–3^. Achieving sustainable groundwater management typically requires reducing extraction and increasing available supply^4^. Managed aquifer recharge (MAR), which uses available water (for example, stormwater, excess flood water and recycled water) and engineered or natural infrastructure for infiltration, can enhance aquifer replenishment by augmenting locally available supplies, thus contributing to an improved water balance^3–9^. Distributed MAR—catchment systems spread throughout a groundwater basin rather than centralized in a single location—holds promise as a low-cost strategy to collect and infiltrate stormwater^5,9–12^. However, distributed MAR also poses unique challenges^4,13–15^. First, infiltration and recharge conditions can be highly heterogeneous, with favourable conditions present across only a fraction of the landscape, including sites located on private land or in areas not accessible to management agencies^16,17^. Second, although components of distributed MAR can be affordable, purchasing or leasing disbursed land assets can be cost prohibitive or politically challenging^11,18–21^.

The objective of this analysis is to introduce recharge net metering (ReNeM), a market-based mechanism that incentivizes groundwater supply augmentation using distributed MAR and to use a case study of ReNeM’s deployment in California’s Pajaro Valley to demonstrate the programme’s cost-effectiveness. Similar to net energy metering, which compensates programme participants with at-home solar power systems for the energy they feed into the grid^22^, ReNeM incentivizes the construction and operation of MAR projects on private property by compensating rechargers based on the measured quantity of water each project infiltrates. ReNeM payments offset direct costs of project operation and land opportunity costs to rechargers—costs that might otherwise be barriers to voluntary action. As currently deployed in the Pajaro Valley, ReNeM engages three key parties: (1) the water management agency that sponsors and manages the programme, approves new projects and issues payments to rechargers; (2) rechargers (for example, landowners or tenants) who facilitate project development and operation on their property; and (3) a third party certifier (TPC) that helps identify viable sites, assists in project design, permitting and construction, monitors water quality impacts and quantifies water infiltrated to inform payments to rechargers. In effect, the TPC augments agency staff and resources while assuring active and prospective rechargers that projects are assessed objectively.

The Pajaro Valley is an agricultural basin on California’s central coast that relies heavily on groundwater to irrigate a variety of high-value crops. Agriculture accounts for approximately 90% of freshwater demand and basin-wide pumping exceeds natural recharge, contributing to chronic overdraft^9^. The Pajaro Valley Water Management Agency (PV Water) is responsible for local groundwater management. PV Water has the legal authority to meter wells that pump more than ten acre feet per year (AFY) (approximately 12,330 m^3^ per year). PV Water is unusual in California in that the agency charges groundwater pumpers a volumetric extraction fee^23^. The extraction fee varies depending on location and availability of alternative water supplies^23^. In many ways, the Pajaro Valley is characteristic of groundwater-dependent agricultural regions that are facing management challenges because supplies are inadequate to meet current and projected demand. The Pajaro Valley’s groundwater basin is one of 127 basins subject to requirements contained in the Sustainable Groundwater Management Act, California’s first state-wide legislation regulating groundwater management.

PV Water’s Board of Directors approved implementation of ReNeM as a pilot programme in 2016, and removed the ‘pilot’ designation in 2021, with a goal of generating an average of 1,000 AFY (1.233 Mm^3^ per year) of infiltration benefit when the programme is fully operational with additional projects^24,25^. Although PV Water and programme participants perceive ReNeM as worthwhile, the programme’s benefits and costs have not previously been quantified. Beyond incentivizing MAR projects that can enhance groundwater supplies for the basin, ReNeM provides additional water quality and non-extractive benefits that can be shared broadly by many groundwater users^26^. Two active ReNeM projects provide the basis for the calculations shown here: Bokariza-Drobac Ranch (BD) and Kelly Thompson Ranch (KT), with 11 and 3 years of operational and cost data, respectively (Supplementary Section 1)^25^. A range of criteria were used to evaluate these sites for inclusion in the ReNeM programme, including infiltration capacity and other hydrologic conditions such as potential water quality impacts. Currently, the University of California, Santa Cruz and the Resource Conservation District of Santa Cruz County jointly act as the TPC. The TPC has accrued high-quality data and samples needed to validate the programme’s functioning during initial ReNeM operations. In the primary cost analyses used in this study, we assume the water management agency directly incurs the TPC costs. As discussed below, we anticipate that the role and form of the TPC may vary if ReNeM is adopted elsewhere.

Annual ReNeM recharger payments in the Pajaro Valley are currently based on a simple formula:

1 $$\mathrm{Payment}={\lambda }_{t}\,{Q}_{t}\,{C}_{t},$$

where *λ*_*t*_ is a scaling factor, *Q*_*t*_ is a project’s net infiltration and *C*_*t*_ is a per unit groundwater pumping fee. Because they are linked to volumetric extraction fees, ReNeM payments are essentially partial rebates, though payments could be linked to other factors instead (Supplementary Section 2). The scaling factor is an agency-established flexible discount mechanism to ensure the programme’s financial viability. Setting *λ*_*t*_ < 1 acknowledges that not all infiltration becomes recharge, not all recharge remains available for recovery and hydrologic accounting involves uncertainty, even with the use of sophisticated tools and methods^27^. Rechargers are paid annually on the basis of the enhanced infiltration generated from their project. Net infiltration—the amount of water infiltrated beyond that which would have infiltrated in the absence of the ReNeM project—is determined empirically using a water balance approach^9^. Water balance calculations account for inflow into dedicated infiltration basins, direct precipitation on the infiltration basin, evaporative losses and changes in infiltration basin storage^9^. For the two sites used as the basis for this analysis, median annual infiltration benefit over their operating lifetimes is expected to be 375 AFY (Table 1).

**Table 1:** Explanation of the variables used for NPV and annualized cost calculations, the values selected for each of these variables and the reasons for selecting those specific values

Variable	Value	Justification for assumed value
Project lifespan (*n*)	25 years	ReNeM was approved initially for 5 years of ‘pilot’ operation^24,48^, but the pilot language was removed in fall 2021 by PV Water’s Board of Directors. For purposes of the CBA, we assumed a 25 year operating period for ReNeM projects.
Quantity of infiltration (*Q*_t_)	375 AFY average (varies)	Estimated total average infiltration for sites BD and KT ReNeM projects is 375 AFY (462,560 m^3^ per year), an average based on hydrologic data from site BD (which has been monitored for 11 years) and runoff estimates for site KT (which has been operating for 3 years).
Water replacement value (*V*_*t*_)	US$650 per AF	A 2014 RCD analysis of site BD estimated that the water replacement value for PV Water in 2012 dollars was US$551 per acre foot (AF) for the first 10 years of the project beginning in 2015, rising to US$2,023 per AF for the next 15 years of the project ending in 2040^49^. RCD’s estimate for the first 10 years corresponded to the average annualized unit cost for a group of low-cost tier projects described in the PV Water Basin Management Plan and visualized in Fig. 1 (ref. ^28^). Adjusting for inflation, US$551 per AF in 2012 amounted to US$650 per AF in 2021. This provided a more conservative estimate than the economically efficient marginal cost.
Volumetric pumping fee (*V*_*t*_)	US$263	The Pajaro Valley’s groundwater pumping fee through 12/1/2022 was US$263 per AF for water users outside the delivered water zone, US$363 per AF for water users within the delivered water zone and US$123 per AF for unmetered rural water users who pump at least 0.5 AF. All ReNeM project sites used in this analysis are located outside the delivered water zone. Pumping fees are periodically updated^50^.
Scaling factor (*λ*)	0.5	ReNeM’s current scaling factor in the Pajaro Valley is 0.5, but it could change in the future.
Discount rate (*r*)	6%	PV Water currently uses a 6% interest rate for financing purposes^28^, which is also near the midpoint of that recommended for agencies by the US Office of Management and Budget^51^. We used this same rate for the CBA.
Number of ReNeM projects (*o*)	2	For initial calculations, we included two operational ReNeM projects.
ReNeM acres (*a*)	10 acres	Approximate area dedicated to ReNeM infiltration at sites BD and KT.
Site management operation and maintenance (*ML*_*t*_)	US$1,000 per acre	When recharge basins are comparable in size to those basins on existing projects, costs are expected to remain relatively linear at a rate of approximately US$1,000 per basin acre per year—an expectation based on actual costs at site BD, assuming 5 person-days of labour per year at a compensation rate of US$100 per hour between 2016 and 2021 (ref. ^49^).
Site supplies operation and maintenance (*ME*_*t*_)	US$500 per project	When recharge basins are fairly close in size to those basins on existing project sites, these costs are expected to remain at US$500 per year for supplies per ReNeM project—an expectation based on annual expenditures at site BD from 2016 to 2021 (ref. ^49^).
Opportunity costs to land (*OC*_*t*_)	US$1,780 per acre	Opportunity cost was based on US Department of Agriculture (USDA) data from 2020 on irrigated land rental rates for Monterey and Santa Cruz counties^52^. As a practical matter, estimates for the value of opportunity costs to land can vary widely. For instance, a case study of site BD estimated an OC of US$7,612 per acre predicated on the assumption that strawberries would have been grown on the land^49^. Contrary to this assumption, at sites BD and KT, zero land was taken out of production to accommodate ReNeM. Therefore, the more modest USDA data was used to estimate OC and the US$1,700 per acre rate was adjusted for inflation. Actual value of land depends on many factors and should be assessed case-by-case.
Fixed project design costs (*F*)	US$847,000	Estimated design and construction costs at site KT were approximately US$750,000, while cost estimates for site BD were approximately US$70,000–100,000 (ref. ^49^). The recharge basin at site BD was constructed before the ReNeM programme, reducing design and construction costs, which only had to fund modification of an existing swale and addition of conveyance infrastructure^9^. By contrast, site KT had to be constructed from scratch. Therefore, this CBA used design and construction costs for site KT as its benchmark. The KT project was funded in 2016 with an approximately US$750,000 design and construction budget—costs that incorporated staff time for permitting—which is equivalent to US$847,000 in 2021.
Annual TPC expenses (*tpcA*_*t*_)	US$13,400 per project	TPC per project costs based on experience with ReNeM in the Pajaro Valley included: consumable supplies, regional transportation for site visits and personnel time to install and recover field instruments, collect and process samples and data, and analyse results and write annual reports.
One-time TPC expenses (*tpcF*)	US$3,700 per project	TPC one-time costs per project included: initial design for monitoring, automated rain gauge with logger, pressure loggers for monitoring inflow and storage, electrical conductivity logger for water quality, and miscellaneous field supplies and hardware.

Where appropriate, costs have been adjusted for inflation (Supplementary Section 7 and Table 3). All variables are shown in italic font.

In this Article, we present a cost–benefit analysis (CBA) that simulates hydrologic variability to model ReNeM and its application in California’s Pajaro Valley^21^. Specifically, we (1) compare ReNeM’s annualized costs with those of other management options^28^, (2) examine ReNeM’s aggregate and distributional net present value (NPV) and (3) consider the sensitivity of ReNeM’s NPV to differences and uncertainties in key variables. Finally, we discuss the implications of these analyses and the applicability of the ReNeM mechanism more broadly.

Three primary results emerged from this analysis: (1) ReNeM has the potential to reduce overdraft at a lower cost than many alternatives under consideration, (2) under various recharge scenarios, ReNeM produces positive net benefits for both the basin and rechargers and (3) ReNeM’s NPV is particularly sensitive to changes in annual recharge yield and the cost of water under alternative strategies.

## ReNeM costs less than other management options

**Fig. 1:**
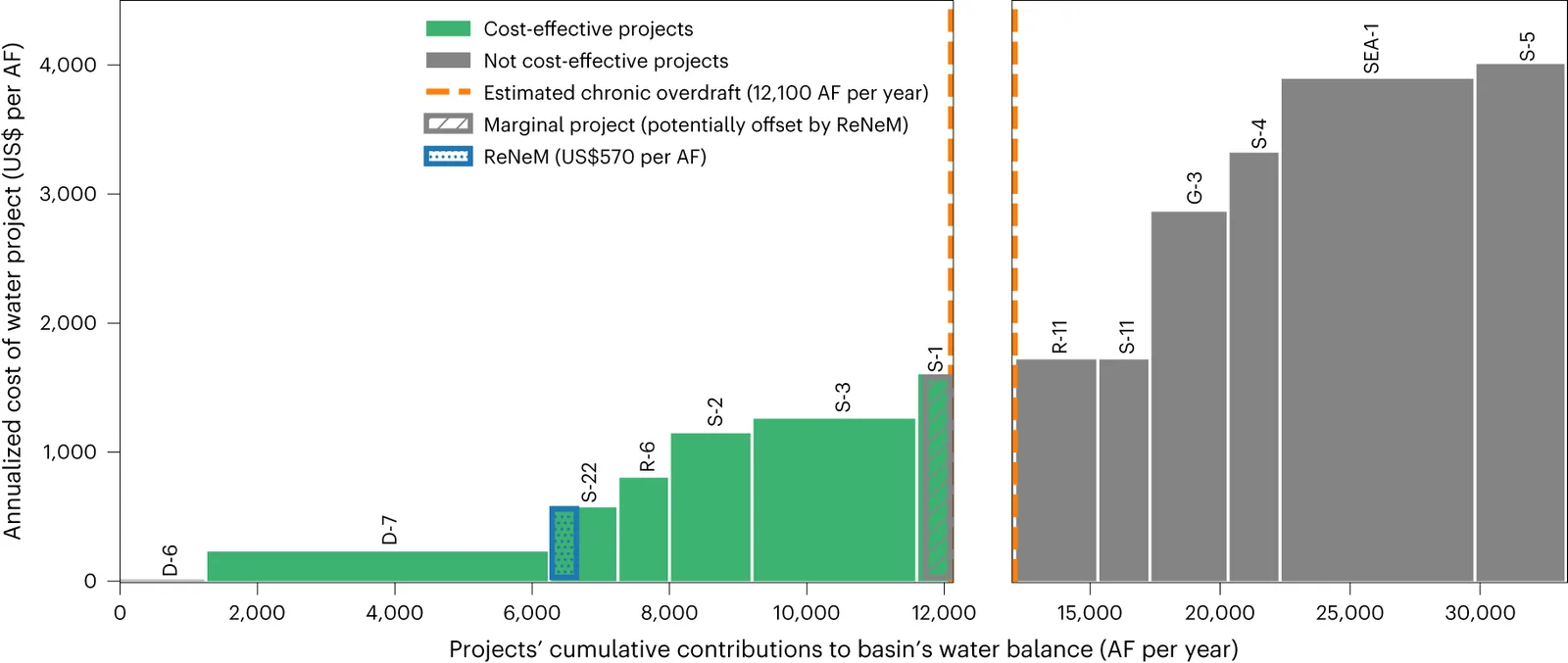
Cost to agency of methods to address chronic overdraft in the Pajaro Valley^53^. ReNeM’s annualized cost compared favourably with that for alternative projects proposed to address chronic overdraft in the Pajaro Valley. At US$570 per AF, ReNeM’s per unit annualized cost was lower than that of all alternative projects except increased recycled water deliveries and conservation. Each block in this figure represents a project that PV Water has under consideration, its estimated cost per AF and its volumetric contribution to addressing chronic groundwater overdraft. The orange vertical line shows the Pajaro Valley’s chronic overdraft volume. Conservation (D-7) is the only demand management strategy prescribed in the BMP. Assuming no other demand management strategies, all projects to the left of this orange line (projects displayed in green) must be implemented to address chronic overdraft. To the right of this orange line, projects in grey were evaluated by PV Water in its BMP, but it considers them to be non-priority projects for addressing the basin’s chronic overdraft. Project codes are defined in Table 2.

Over a 25 year period, two ReNeM sites in the Pajaro Valley (BD and KT) are expected to collectively generate 9,375 AF (approximately 12 Mm^3^) of infiltration benefit, at a cost that is favourable compared with other available management options (Fig. 1 and Table 2). The full suite of project options ranges in cost from US$0 per AF for enhanced recycled water deliveries to US$7,500 per AF for desalination, with ReNeM falling towards the lower end of the range at US$570 per AF (Table 2). In addition, while ReNeM cost data and volumetric contribution data (Fig. 1 and Table 2) were based on direct observations at two operating sites, cost estimates for other projects available to PV Water were projections, leaving open the possibility that cost overruns could increase expenses for other options, which is not uncommon for infrastructure projects in the water sector.

**Table 2:** A comparison of the annualized cost (2021 US$) and yield estimates for ReNeM and for PV Water’s priority projects, as identified in its 2014 BMP

Water management project and description	Cost/AF (2021 US$)	Yield estimate (AFY)
**(D-6) Increased recycled water deliveries** Increase night-time irrigation season and shoulder period recycled water deliveries	US$0	1,250
**(D-7) Conservation** Improvements in irrigation efficiency	US$229	5,000
**ReNeM** Incentives for private land use practices that encourage recharge. Programme goal is to achieve 1,000 AFY, but only 375 AFY is modeled in this Article	US$570	375
**(S-22) Harkins Slough recharge facility upgrades** Infrastructure improvements to achieve maximum allowable project yield. Diversion of water for recharge during winter and subsequent extraction during summer for irrigation	US$572	1,000
**(R-6) Increased recycled water storage at treatment facility** Construction of storage facilities to provide additional day-time deliveries of recycled water for irrigation	US$801	750
**(S-2) Watsonville Slough with recharge basins** Construction of facilities and infrastructure necessary for diversion of water for recharge during winter and subsequent extraction during summer for irrigation	US$1,145	1,200
**(S-3) College Lake with inland pipeline to Coastal Distribution System** Summer diversion of water from College Lake and Pinto Lake via pipeline for irrigation	US$1,259	2,400
**(S-1) Murphy Crossing with recharge basins** Diversion between December and May of the Pajaro River at Murphy Crossing to nearby infiltration basins	US$1,602	500
**(R-11) Aquifer storage and recovery** Diversion of water from Corralitos Creek, Pinto Lake and Watsonville Slough for injection during the winter and extraction during the summer	US$1,717	3,200
**(S-11) River conveyance of water for recharge at Murphy Crossing** Conveyance of Central Valley Project contract water from the Mercy Springs Water District via a pipeline from the Santa Clara Conduit to the Pajaro River for release and recharge at Murphy Crossing during low-flow months	US$1,717	2,000
**(G-3) San Benito County groundwater demineralization at Watsonville wastewater treatment plant (WWTP)** Treatment of San Juan Valley groundwater at the Watsonville WWTP for high levels of total dissolved solids to reduce these levels	US$2,862	3,000
**(S-4) Expanded College Lake with Pinto Lake, Corralitos Creek, Watsonville Slough and aquifer storage and recovery (ASR)** Increase College Lake’s storage capacity and its water supply and develop seasonal storage using ASR	US$3,319	2,000
**(SEA-1) Seawater desalination** Construction and operation of a seawater desalination facility	US$3,892	7,500
**(S-5) Bolsa de San Cayetano with Pajaro River diversion** Dam and reservoir construction for seasonal surface water storage of Pajaro River flows and recycled water	US$4,006	3,500

Project codes included in parenthesis correspond with those used in PV Water’s 2014 Basin Management Plan (BMP). Project titles and descriptions synthesize information also available in PV Water’s 2014 BMP. These data illustrated ReNeM’s cost effectiveness on a per acre foot basis—a key point visualized in Fig. 1 and further emphasized by Supplementary Table 4, which details the range of management projects PV Water omitted from consideration owing to, among numerous other reasons, high cost, insufficient yield and political unacceptability. All costs are adjusted for inflation (Supplementary Section 7 and Table 3).

Results substantiate PV Water’s expectation that ReNeM’s costs are ‘favourable compared with the cost of alternative water supplies’^24^, making ReNeM a financially attractive option.

## Costs and benefits vary for cooperating parties

**Fig. 2:**
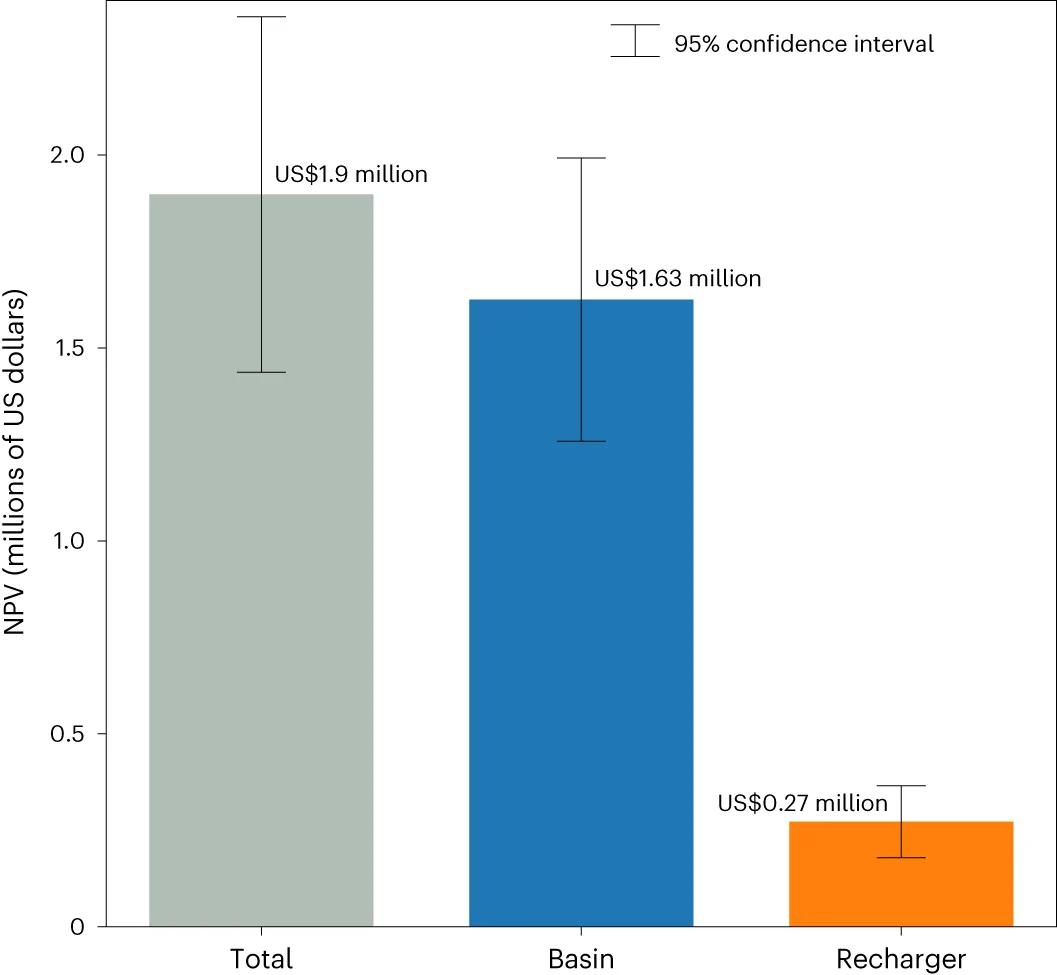
NPV distribution. ReNeM produced a NPV of $1.9 million over a 25 year time horizon. This NPV is distributed between rechargers and PV Water on behalf of the groundwater basin to the tune of US$270,000 and US$1.63 million, respectively. Error bars show 95% confidence intervals based on a Monte Carlo simulation that modelled the impact of hydrologic variability on infiltration.

ReNeM depends on multiple parties cooperating towards a common goal. We disaggregated ReNeM’s NPV to examine the distribution of costs and benefits among parties (Fig. 2). Under all scenarios examined, ReNeM produced notably more benefits than the costs it imposed. With two projects (BD and KT) operating for 25 years, ReNeM is projected to generate net benefits equivalent to approximately US$1.9 million, with approximately 86% of that amount (US$1.63 million) accruing to the basin as resource enhancement and approximately 14% (US$270,000) paid to programme participants who host projects on their land. The benefits of ReNeM were calculated on the basis of the replacement value of water, for which we used a conservative estimate of the unit cost of water provided by other infrastructure projects and management options (Fig. 1 and Table 1). Because ReNeM offsets more expensive capital investment projects, it has a positive NPV, whereas the most expensive management options have a negative NPV^28^.

## Sensitivity analysis highlights benefit and adaptability

**Fig. 3:**
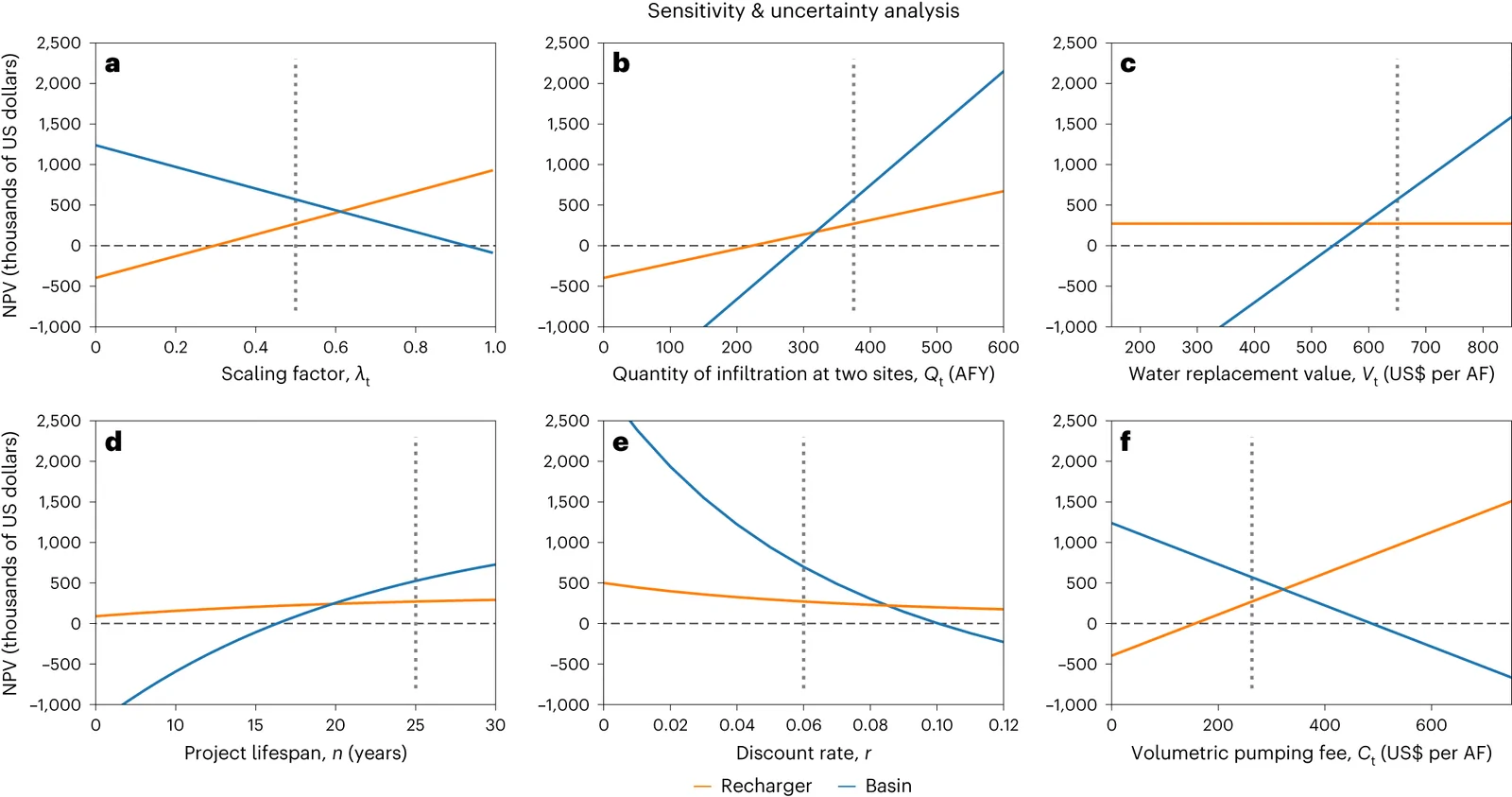
The distribution of costs and benefits. **a**–**f**, The distrbution was found to be sensitive to key variable inputs including scaling factor (**a**), annual recharge yield (**b**), water replacement value (**c**), project lifespan (**d**), discount rate (**e**) and volumetric pumping fee (**f**). Crucially, the scaling factor in **a** is a policy variable that can be adjusted by the agency to achieve the desired distribution of benefits and account for hydrologic uncertainty.

A sensitivity analysis highlights ReNeM’s adaptability and the wide range of scenarios under which ReNeM could be economically viable (Fig. 3). Some variables’ values depend on well-established empirical data (for example, operation and maintenance costs and TPC costs), whereas others rely more strongly on economic assumptions (for example, water replacement value).

The scaling factor (*λ*_t_)—a discretionary discount factor set by agency personnel—was a crucial element in this analysis, through which managers could adjust the expected distribution of benefits among parties. At present, with λ_t_ = 0.5, more benefit goes to the basin than to individual rechargers (Fig. 3b). Improved project performance, higher water replacement value and longer project lifespan (Fig. 3b–d) all tend to proportionately favour the basin. That said, more infiltration per project would increase the programme’s aggregate NPV and its NPV for both the basin and rechargers, an alignment of interests that highlights ReNeM’s collaborative potential. By contrast, a higher discount rate or higher pumping fee (Fig. 3e,f) tend to offer proportionately greater benefits to rechargers, although the latter also increases overall water costs.

**Fig. 4:**
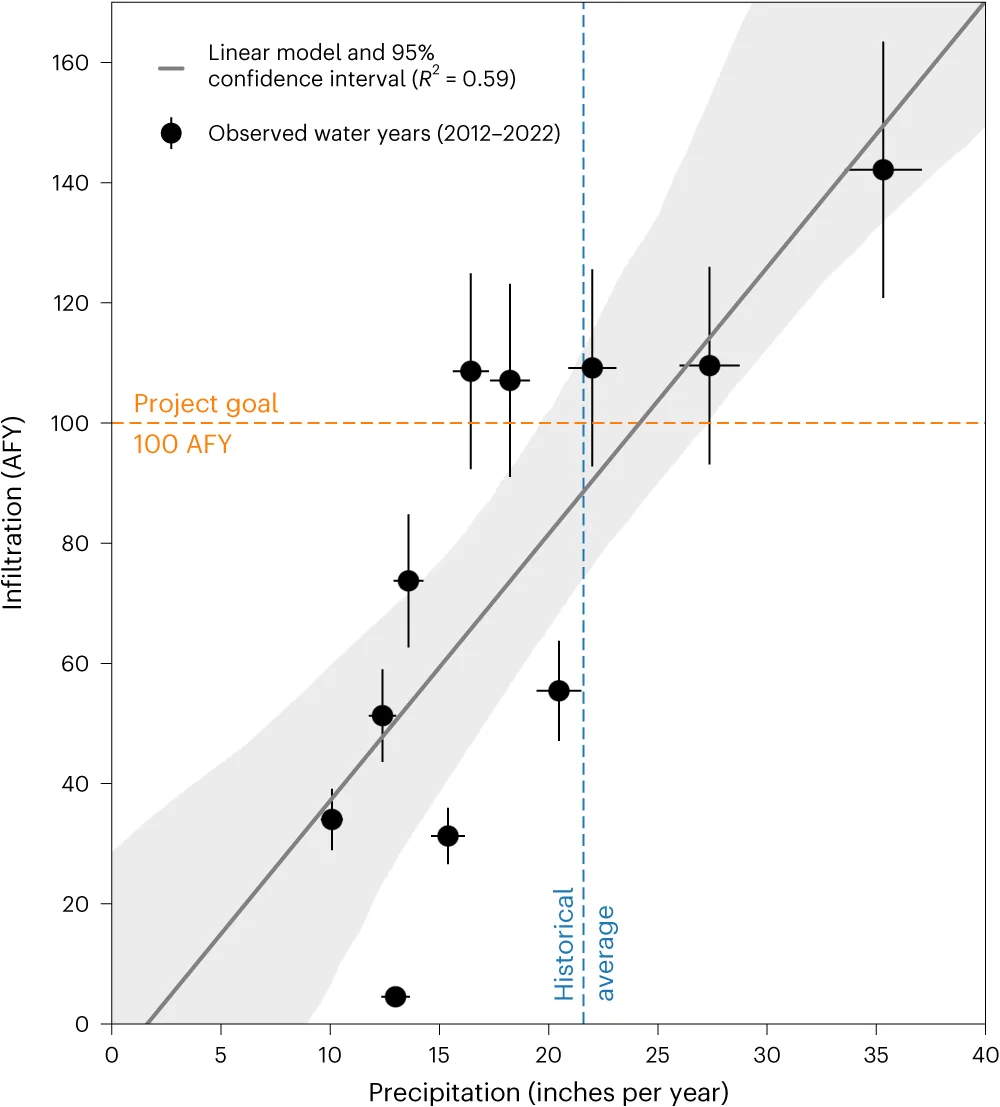
Runoff data for ReNeM’s BD project. There is a linear relationship between annual precipitation and infiltration from runoff. Precipitation and infiltration data are available for the BD ReNeM project site for water years 2012–2022.

As with other water management strategies, agencies and rechargers participating in ReNeM must accept the implications of hydrologic variability. These implications include the possibility that severe drought can produce negative economic returns for one or more years (Fig. 3b), even if the average return is positive over a period of decades. During drought conditions with little infiltration, rechargers may incur maintenance and opportunity costs that the performance-based payment model will not fully offset. In applying an analysis of this kind, water managers must decide how they wish to estimate hydrologic conditions in the future. For the present analysis, we applied a runoff response from one ReNeM site (BD) for which there are 11 years of direct observations (Fig. 4), and assumed a proportionate response at the second site (KT). Estimates of this kind will be refined as more data are collected at these sites, and as additional ReNeM sites are added. Although ReNeM offers promising long-term economic viability, risk aversion and rechargers’ annualized budget expectations highlight the need to address limited benefits that may be generated over the short term (Supplementary Section 3 and Figs. 1–3).

## Broader potential and importance of ReNeM

ReNeM has been cost effective in the Pajaro Valley. Although it remains to be seen how the programme could be modified and applied in other settings, these results suggest that this mechanism may be cost effective elsewhere. PV Water plans to expand recharge infrastructure at several existing sites. In addition, ReNeM can stimulate further expansion of MAR by increasing access to land with appropriate hydrology (for example, sufficient runoff, acceptable water quality, good infiltration conditions and space in underlying aquifers), including private property. ReNeM’s costs are anticipated to scale as more projects are added.

ReNeM presents an alternative mechanism whereby stakeholders are financially incentivized to conduct recharge on their land. Globally, groundwater management strategies range from market-based instruments such as pumping fees to more prescriptive policies such as pumping quotas^29^. However, political factors and transaction costs associated with collective action can hinder effective groundwater management^30,31^. ReNeM may present a promising strategy to cost-effectively manage groundwater by subsidizing recharge, ideally as a complement to other approaches, including demand management^32^. Crucially, the TPC has absorbed and mitigated transaction costs during ReNeM’s initial operation in the Pajaro Valley. However, we have shown that even if the agency internalized these costs, the programme would remain cost effective.

Key parameters controlling the distribution of ReNeM costs and benefits can be adjusted to account for local conditions and preferences and to re-distribute risk. Changing the scaling factor (*λ*_*t*_) can reallocate benefits and address programme participation needs (Fig. 3e)—needs potentially influenced by an agency’s financial or capacity limitations or a desire to encourage more participation in the programme. Broad cost categories such as design, construction and TPC services can also be allocated differently to alter which ReNeM party—rechargers, the agency or others—internalizes specific costs (Supplementary Section 4 and Figs. 2 and 4). This adaptability also means agencies could link ReNeM payments to groundwater pumping fees or adapt the programme to link payments to other benchmarks such as the market cost of groundwater, value of land, or other economic drivers.

ReNeM may provide other benefits beyond those monetized in this CBA, which could motivate operation of ReNeM programmes in the Pajaro Valley and elsewhere^33^. Unlike centralized groundwater banking programmes, ReNeM does not confer a right to the later withdrawal of infiltrated water^21^. For this reason, ReNeM is well positioned to augment baseflow in regional streams, improve water quality by diluting salts and nutrients, and enhance aquatic conditions for the benefit of the basin and its ecosystems^34^. With appropriate enabling conditions such as groundwater protections or extraction limits, ReNeM gives agencies flexibility to balance a variety of water uses, exercise demand management tools to limit withdrawal of recharged water, and provide flexible support for groundwater users.

In addition to the programme’s favourable cost profile (Fig. 1 and Table 2), ReNeM’s incentive-based management represents a qualitatively different mechanism from other groundwater management strategies. This performance-based compensation system aligns agency and recharger incentives, since both benefit from land use practices that promote infiltration when water supplies are available. Rechargers receive greater financial payout for better project performance, the agency enhances groundwater conditions in a cost-effective way, and the basin benefits from improvements to the shared resource. To the extent that it helps to bring a groundwater basin back in to hydrologic balance, thereby achieving sustainability goals that can generate improvements to baseflow or reducing seawater intrusion. ReNeM falls within a broader class of incentive-based policy instruments intended to provide environmental and ecosystem services in the absence of existing markets^18,35,36^. In this sense, ReNeM can also align incentives between a public agency responsible for environmental stewardship and private landowners who benefit from sustainability of groundwater supplies and associated environmental improvements because both benefit financially from the infiltration ReNeM incentivizes^18,37,38^. ReNeM compensates rechargers based on the amount of additional infiltration the projects generate—infiltration that accrues to the groundwater basin. Under this outcomes-based mechanism, rechargers must balance the value of additional payments they will receive if they practise particular land management strategies that promote groundwater recharge at their project sites (for example, use of cover crops to maintain open soil structure during the dry-season, vegetation removal to reduce evapotranspiration losses before the wet season and scraping and discing of infiltration sites to enhance recharge rates) with the costs of those management practices.

ReNeM is probably best suited for implementation as part of a broader portfolio of management strategies. ReNeM’s performance-based payments are a supply-side corollary to demand-side pumping fees. This makes ReNeM well suited for use in conjunction with demand-side management that levies extraction fees on groundwater users; payments can be pegged to extraction fees which can in turn make the programme revenue-neutral, or even revenue-positive for the agency.

Ample literature underlines the value of broad stakeholder involvement for ensuring robust management strategies that enjoy widespread support^39,40^. Helpfully, ReNeM’s structure is inherently participatory, relying on voluntary cooperation between rechargers and an agency to launch and manage individual projects, with TPC services providing the programme support to help rechargers understand the ramifications of different land management strategies. This multi-party cooperative framework has advantages not offered by most centralized strategies. ReNeM directly engages resource users in groundwater recharge and sustainable water management, supporting the broader goal of hydrologic balance^32,36,41,42^. For instance, in the course of investigating the physical characteristics of potential project sites, the ReNeM programme incorporates outreach with many resource users, a process that can benefit communication among stakeholders about sustainable groundwater management. Some of these resource users go on to steward ReNeM projects. Outreach to and collaboration with resource users can increase awareness of groundwater issues, in turn producing both supply-side and potential demand-side benefits where informed resource users adjust consumption practices^43,44^. Of course, in addition to satisfying fiscal constraints, ReNeM must operate within other relevant constraints such as permitting requirements, zoning law and local political and social influences.

In light of near-universal groundwater mismanagement, the world is thirsty for innovative practices that can contribute to sustainable groundwater management. The analysis we present demonstrates ReNeM’s cost effectiveness for the range of parties that collaborate on this distributed solution in the Pajaro Valley. Water managers can use the methodology presented here to assess whether ReNeM may be viable in their own management areas, to determine economic conditions under which an incentive-based recharge programme holds the most promise, and to design an analogous incentive-based recharge programme tailored to local conditions. To the extent that it can generate novel incentives for sustainable management to the Pajaro Valley and other basins around the world, ReNeM contributes a new option that agencies can use to address the global groundwater crisis.

## Methods

CBA is a tool for comparing the cost effectiveness of various policy options^45^. This CBA (1) calculated and compared ReNeM’s annualized costs with the annualized costs of alternative methods that PV Water has identified as part of basin management planning^28^. It then (2) calculated NPV of ReNeM’s benefits and costs to (3) assess how these costs and benefits were distributed between participating parties. Finally, it (4) used a sensitivity analysis of model parameters to understand the range of conditions under which ReNeM’s NPV remained positive. All data generated or utilized by this analysis are included in the published article and its Supplementary Information.

### Cost comparison with other water management options

The first portion of this CBA used an annualized cost analysis to examine how ReNeM’s costs compared with the costs of alternative water management methods. The annualized cost captured a project’s cost per year over that project’s lifespan and enabled a comparison of annual project costs for projects with different lifespans. The formula for annualized cost used a discount rate (*r*), a project lifespan (*n*) and an NPV estimate of projected costs (*K*). The formulas for *K* and annualized cost are

2 $$K=\mathop{\sum }\limits_{{t}=1}^{n}\frac{{\mathrm{costs}}_{t}}{{(1+r)}^{t}}$$

3 $$\mathrm{Annualized}\,\mathrm{cost}=\frac{K\times r}{1-{(1+r)}^{-n}}$$

ReNeM’s costs included (1) operation and maintenance costs such as equipment, labour, permitting (Supplementary Section 5) and TPC services, (2) capital costs such as design and construction and (3) the opportunity cost of land used for recharge. ReNeM’s costs also included transaction costs associated with finding a TPC, landowner outreach and overall programme management, although these costs were unable to be quantified in the Pajaro Valley and therefore not included in this analysis. Importantly, the cost of water supply to PV Water’s ReNeM programme was zero because rechargers used hillslope runoff. However, other locations that explore a ReNeM programme may use hillslope runoff with associated permitting costs or alternative water supplies with associated costs. In either case, these locations will want to incorporate the cost of water into an economic analysis of the program.

### NPV analysis

Our CBA also quantified the magnitude and distribution of costs and benefits between rechargers, the agency operating on behalf of the groundwater basin, and the TPC. The basis for the portion of this CBA that estimated ReNeM’s distributional implications relied on NPV. NPV is an estimate of a project’s return on investment over the project’s lifespan discounted to the present and was helpful for comparing the distribution of net benefits between different parties. NPV also used a discount rate (*r*), a project lifespan (*n*), and projected benefits and costs for each annual time step (*t*), calculated here as:

4 $${\mathrm{NPV}}=\mathop{\sum }\limits_{t=1}^{n}\frac{{\mathrm{benefits}}_{t}-{\mathrm{costs}}_{t}}{{(1+r)}^{t}}.$$

ReNeM’s primary monetized benefit was the money saved by not undertaking other, more costly groundwater management projects required to correct overdraft. ReNeM and alternative groundwater management strategies all achieved the same benefits associated with correcting overdraft and raising the groundwater table. As such, this cost–benefit analysis of ReNeM relative to an alternative groundwater management strategy could also be considered a cost-minimization exercise or cost-effectiveness analysis. ReNeM’s ‘benefits’ were quantified as the programme’s relative cost savings, which was calculated by multiplying the amount of water infiltrated by the programme by the water replacement value—a proxy for the cost of obtaining water via some other supply-augmentation or demand-reduction groundwater management measure, as captured by the capital cost of water projects, generally (Fig. 1). After calculating the programme’s overall NPV, this CBA then considered how ReNeM’s benefits and costs were distributed separately between rechargers and the agency by allocating certain costs to different parties and calculating the rebate payments paid to rechargers under particular infiltration scenarios.

### Estimating infiltration volume

ReNeM’s rebate payment—the benefit rechargers receive and cost the agency incurs—was linked quantitatively to annual infiltration volume. Site selection, system design and project management decisions can influence a project’s performance, but the volume of infiltration also depends on hydrologic conditions, which vary on intra-annual and inter-annual timescales. The distribution of annual infiltration volumes across the project lifespan impacted the NPV of ReNeM. To estimate the influence inter-annual hydrologic variability had on the potential spread of ReNeM projects’ benefits over time and thus their NPV, we ran a Monte Carlo simulation in which infiltration volumes were sampled from a time series of historical estimates. This approach modelled both the range of hydrologic conditions and the possible sequencing of wet and dry years to produce both the distribution and probability of potential outcomes (Supplementary Section 6, Figs. 5 and 6 and Tables 1 and 2) to simulate annual hydrologic variability at the two existing ReNeM sites.

Annual infiltration parameters for the Monte Carlo simulation were generated using 20 years of recorded monthly data from a nearby rain gauge, extrapolating infiltration from rainfall data. For purposes of this CBA, we applied a linear relation between (1) precipitation measured by a local rain gauge and (2) resulting runoff that flows into an infiltration basin, the vast majority of which becomes infiltration^9^. This relationship was scaled to represent the estimated total average infiltration from two of ReNeM’s operating projects (sites BD and KT) of 375 AFY (462,560 m^3^ per year), resulting in the relationship

5 $${Q}_{\mathrm{{est}}}=P\times X ,$$

where *Q*_est_ is estimated annual infiltration in AF, *R* is annual rainfall in inches and *X* is a runoff collection factor—the slope of the line relating *P* to *Q*_est_ (*X* = 18 in this analysis). This linear relationship was developed on the basis of more than 10 years of detailed, empirical data from site BD that were used to derive a functional relationship between rainfall and collected runoff (Fig. 4) and rational runoff models generated by Natural Resources Conservation Service for the KT project site. Of course, the relationship between runoff and infiltration can vary substantially, depending on numerous factors beyond annual rainfall, including, though not limited to, the intensity and persistence of storms, antecedent soil moisture conditions and geology. Although other basins and potential project sites may be assessed with more sophisticated predictions of runoff generation, this simple approach offered an opportunity for a rapid assessment of a range of conditions, as observed at an operating ReNeM site. Many water management agencies have practical runoff models or other predictions they use for a variety of purposes, and those could be employed for ReNeM siting assessments. The actual relationship between rainfall and infiltration is certainly more complex, but (1) a simple approach based on widely available data such as monthly precipitation allows for initial application to other settings, and (2) this approach can readily incorporate hydrologic variability as part of the Monte Carlo analysis. All such relations will require updating in response to ongoing and future climate change.

### Parameter values and economic assumptions

This economic analysis was based on the following approach and assumptions. We assumed that many of the variables used to calculate NPV had fixed values over the period of analysis, nominally 25 years, and we estimated these values based on ReNeM operations in the Pajaro Valley (Table 1). The sensitivity of selected NPV estimates to changes in these and other parameter values was also explored (Fig. 3).

We also assumed that PV Water, acting on behalf of the basin, would pursue some type of groundwater management or water procurement strategy, as opposed to a no-action alternative in which groundwater sustainability concerns were not addressed. This was a reasonable assumption under Californian law^46^. We assumed that, in the absence of ReNeM, rechargers would put the portion of their land dedicated to recharge to another economically productive use.

### Reporting summary

Further information on research design is available in the Nature Portfolio Reporting Summary linked to this article.

## Supplementary information


Supplementary InformationSupplementary Figs. 1–6, Discussion and Tables 1–4.
Reporting Summary.


## Data Availability

All data generated or utilized by this analysis are included in the published article and its Supplementary Information.

## Code availability

Code files can be accessed at repositories under the GitHub account in ref. ^47^.
