# Supplementary material for: Recharge net metering (ReNeM) is a novel, cost-effective management strategy to incentivize groundwater recharge
Source: Nat Water. 2023 Oct 18;1(10):855–63. doi: 10.1038/s44221-023-00141-1 (PMC13533828; doi:10.1038/s44221-023-00141-1)
Supplement: Supplementary file 1 [file 44221_2023_141_MOESM1_ESM.pdf]

nature water

Article https://doi.org/10.1038/s44221-023-00141-1

Recharge net metering (ReNeM) is a novel, cost-effective management strategy to incentivize groundwater recharge

In the format provided by the authors and unedited

SUPPLEMENTAL INFORMATION

1. KT and B project background and ReNeM site selection

The Bokariza project was incorporated into the ReNeM program in the Pajaro Valley during the program's first operating year. The Bokariza project collects runoff from a ~170 acre drainage area using a system of culverts, a sediment detention basin, and a ~4 acre infiltration and recharge basin.[9] The Bokariza infiltration basin was created initially by growers prior to the launch of the ReNeM program, by modifying a natural swale in the landscape, mainly in an effort to limit runoff and flooding of adjacent properties during large storms,[9] and was subsequently modified with a larger inflow culvert, dedicated sediment detention basin, and more extensive network of ditches for stormwater collection. The Kelly Thompson infiltration and recharge system was constructed in Fall 2019 and has operated as part of the ReNeM program for three years to date. This project is located on a parcel that was previously farmed, collecting hillside runoff from ~1,300 acres of rangeland and crops, and routing water through a ~1 acre sediment detention basin and into a ~4 acre infiltration and recharge basin.

Bokariza and Kelly Thompson ranches are actively farmed for crops including strawberries, cane berries, and salad vegetables.[9] Both project sites collect excess hillslope runoff during heavy rain events and divert these flows into dedicated infiltration basins. These are flows that in past years would have passed into the Pajaro River and to the adjacent Monterey Bay (Pacific Ocean) if they were not slowed so that some infiltration could occur. A study of the Pajaro Valley suggests that agricultural and urban activity have substantially increased groundwater extraction while at the same time reducing natural infiltration for groundwater recharge.[53] ReNeM projects serve to recreate a portion of the region's hydrologic system services that have been lost because of human modifications to the landscape and to the water cycle.[2]

ReNeM site selection and project development followed a plan generated for the program by the TPC that was structured as a three-stage process based on a series of agreements, available at the hyperlink: https://www.rcdsantacruz.org/managed-aquifer-recharge. Prior to this three-stage process and prior to program launch, TPC members described ReNeM at public meetings, using maps that included a preliminary recharge suitability assessment as visual aids so that prospective rechargers and other interested parties could identify locations of interest.[16] Other prospective rechargers have called or sent emails asking that particular sites be considered for inclusion in ReNeM—often on properties they own or farm (most growers in the Pajaro Valley being tenants). Prospective rechargers were asked to complete a Stage 1 ReNeM agreement, "Landowner Request for Assistance." This document explains a bit about the program, gives the TPC permission to visit and examine the site, and describes issues involving risk and confidentiality. Initial considerations for site selection included site access, hydrologic conditions, drainage area, evidence for runoff generation, current land use, and extent of landowner and tenant interest. The TPC could also consider the locations of other project sites in the region, as it can be desirable to have these spatially distributed.

If a site looked promising, a prospective recharger was asked complete a Stage 2 agreement for property access. This document explains more about how ReNeM works and provides permission for a more invasive site evaluation, potentially to include soil and runoff sampling and subsurface assessment with remote methods and/or drilling. Additional documentation was required, including a property deed, terms for an associated grant if relevant, expected operation and maintenance steps, and regulatory-based reporting requirements. Finally, if a project was selected to move forward as part of the ReNeM program, the TPC and recharger completed a Stage 3 agreement, which sets terms for project development, operation, and long-term monitoring for physical performance and impacts to water quality. There may still be a need to raise capital funds or clear other hurdles, and this agreement provides confidence that it will be worth expending effort towards project implementation.

2. Rebate benchmark

PV Water links rebate payments to volumetric extraction fees. Pajaro Valley is unusual because it has groundwater extraction fees that are large relative to existing fees in other areas. Other basins interested in deploying a ReNeM-like program that do not have groundwater extraction fees have at least two options First, agencies can implement volumetric fees in their service area. While doing so will often be politically challenging, a ReNeM-like program has significant potential and the rebates it generates for some water users can ameliorate the challenges of a fee proposal. Second, where extraction fees are infeasible or insufficiently large to spur interest in ReNeM among water users, alternative frameworks could be developed that link rebate payments to other benchmarks.

3. Baseline payment

PV Water currently employs a per-project rebate formula of $Payment = \lambda_t Q_t C_{t,}$, where λ_t is an agency-established scaling factor, Q_t is a project's net infiltration, and C_t is a per-unit groundwater pumping fee, as described in the main text of this paper. However, some stakeholders considering participation in ReNeM have requested measures to address the risk they incur during dry years with no infiltration. In response, the agency is considering incorporation of a baseline payment that would update this formula to $Payment = \lambda_t Q_t C_t + s_t$, where s_t is a baseline payment. This baseline guarantees rechargers a minimum annual payment, regardless of hydrologic conditions or project performance.

The incorporation of baseline costs is meant to reduce the risk dry years pose to participation. At present, payments to ReNeM participants are fully dependent on project performance. In drought years such as the Pajaro Valley has recently experienced, projects generate relatively little infiltration. ReNeM participants who maintained runoff routing and infiltration systems may have incurred operations and maintenance costs not fully offset by rebate payments. To address this perceived risk as a potential barrier to program participation, the ReNeM formula can be adapted to add a baseline payment to the performance-based rebate payments. Results of our analysis show that, even with a minimum guaranteed annual baseline payment of $5,000/site which allocates a greater portion of the ReNeM program's benefits to participants, the basin's distribution of NPV remains positive (SI Fig. 3). Inclusion of a baseline payment does not significantly increase ReNeM's cost to the agency, and ReNeM remains cost-effective relative to other groundwater management options (SI Figs. 4 & 5). Sensitivity analysis results also indicate that this baseline payment has room to expand significantly without causing agency/basin costs to exceed agency/basin benefits (SI Fig. 6).

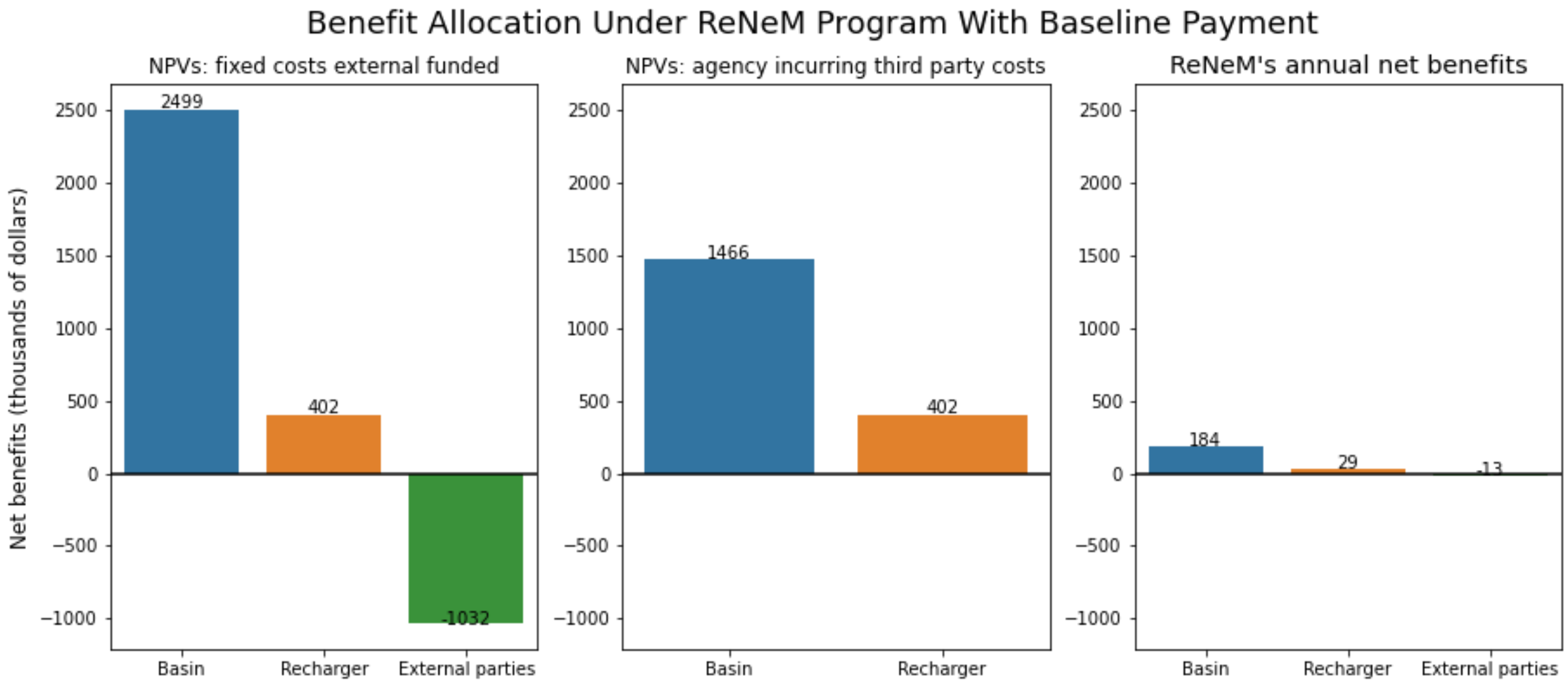

SI Figure. 1: Over its lifetime, a ReNeM program that incorporates a baseline payment of $5,000 to rechargers still produces substantial net benefits for both rechargers (orange) and the basin (blue), regardless of whether the agency acting on behalf of the basin internalizes TPC expenses (green).

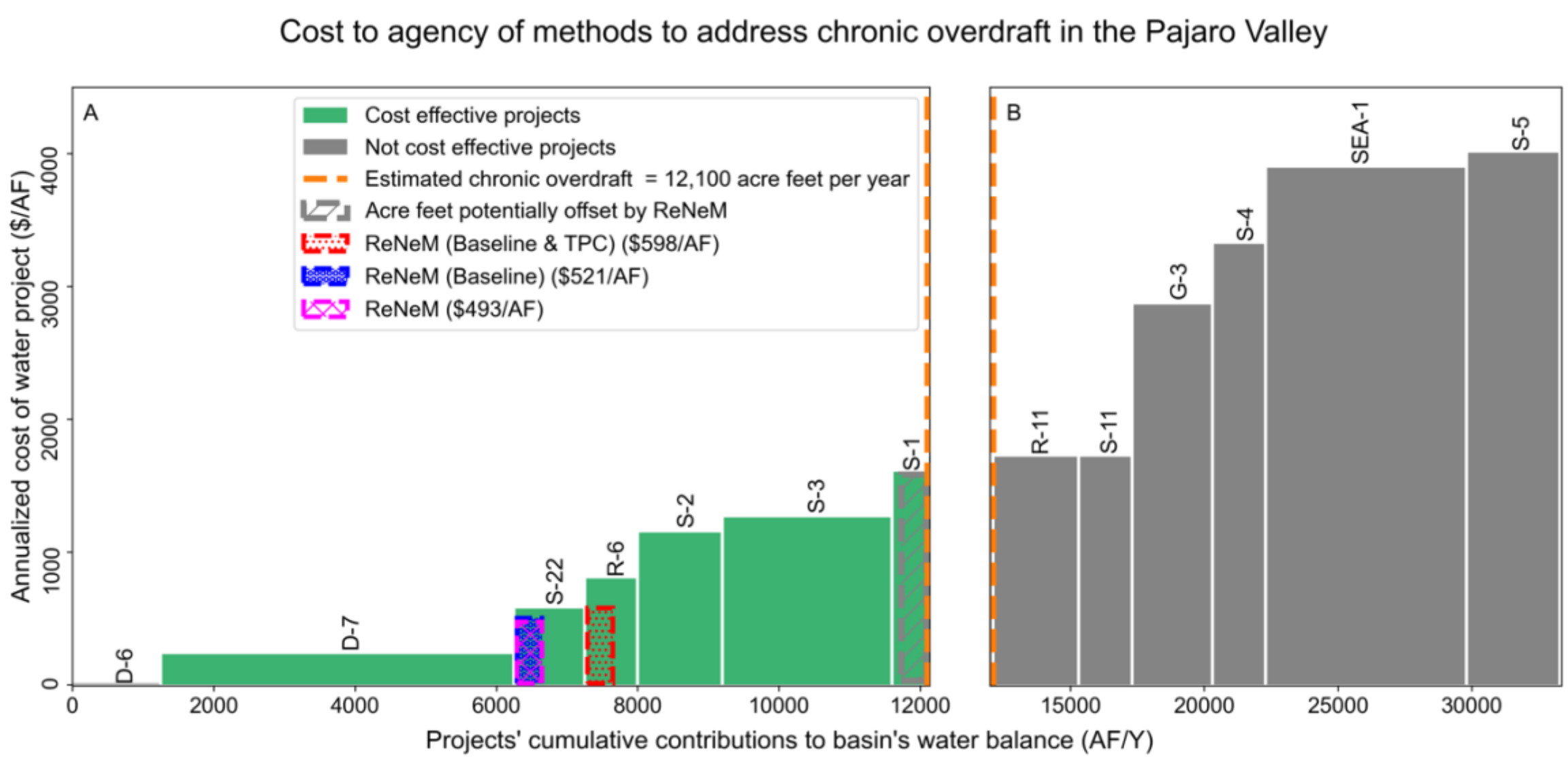

SI Figure 2: ReNeM's annualized cost compares favorably to that for alternative projects proposed to address chronic overdraft in the Pajaro Valley. At $493 per AF, ReNeM's per unit annualized cost is lower than that of all alternative projects except increased recycled water deliveries and conservation. Even when rechargers receive baseline payments, at $521 per AF, ReNeM's per unite annualized cost is low. When rechargers receive baseline payments and the agency internalizes TPC expenses, at $598 per AF, ReNeM's per unit annualized cost continues to compare favorably and is lower than that of all alternative projects except increased recycled water deliveries, conservation, and Harkins Slough recharge facility upgrades. The orange vertical line shows chronic overdraft volume. Assuming no demand management strategies, all projects to the left of this line must be implemented to address chronic overdraft. Project codes are defined in Table 1.

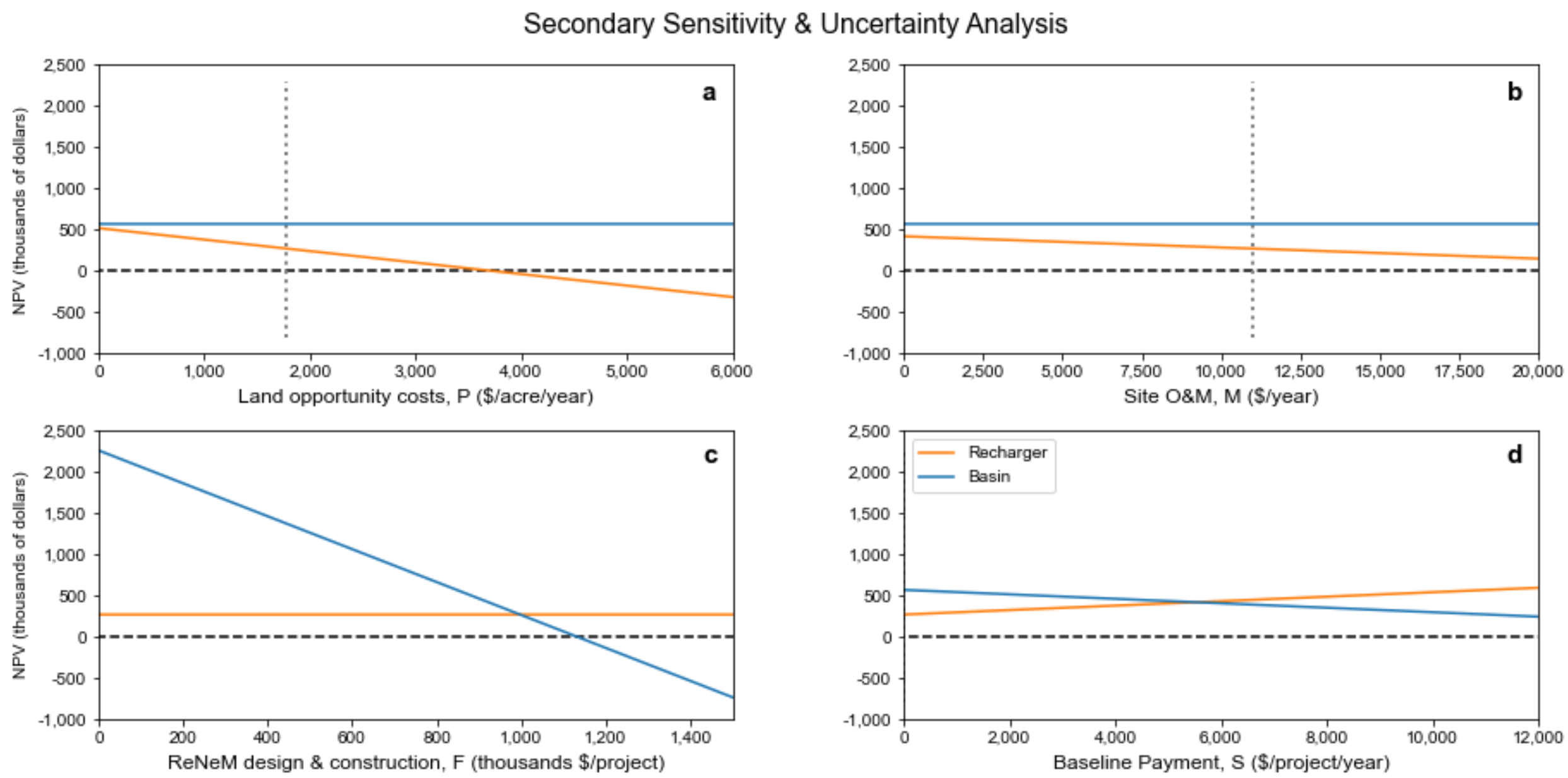

SI Figure 3: The distribution of costs and benefits may also be sensitive to other variable inputs (TPC costs, opportunity costs, baseline payments, operation and maintenance, and design and construction). The baseline payment in particular is a variable the agency can choose to incorporate and adjust to achieve its desired level of recharger participation and distribution of benefits.

The preceding analysis suggests that the economics of a baseline payment may work for all parties. Further analysis would be needed to assess whether inclusion of a baseline payment is necessary to ReNeM's successful deployment. Remaining questions about this aspect of program design include whether it makes the program more palatable to current and prospective rechargers while still providing a cost-effective program with positive NPV for all participants over the modeled project period, and what the optimal size of the baseline payment would be.

4. Externally-funded TPC expenses

Pajaro Valley's ReNeM program relies on third party certification of projects—a process that imposes costs. The Pajaro Valley has successfully sought external funding for TPC services. It has also used this external funding to finance portions of project design and construction. This has reduced both recharger and agency expenses (see SI Fig. 1). However, the body of this analysis models scenarios under which PV Water internalizes TPC expenses and rechargers fund capital costs in order to mimic a ReNeM program that is self-supporting. It is possible that other agencies interested in deploying ReNeM can fund certain program expenses externally. Under these circumstances, it may also be possible for ReNeM's cost per AF to drop below the cost per AF to pump groundwater, making the ReNeM program revenue-positive for the agency. Importantly, external funding of TPC services influences the distribution of ReNeM's benefits (SI Fig. 2).

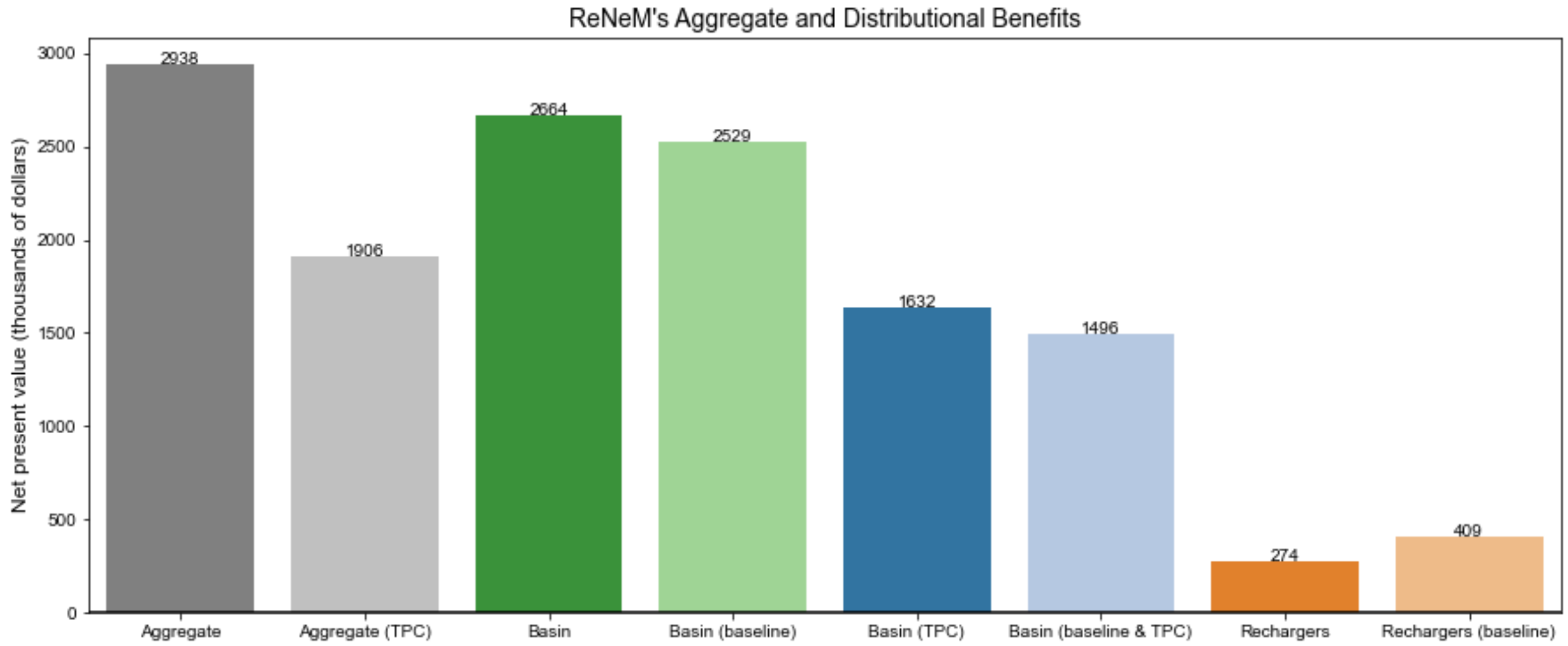

SI Fig. 4: ReNeM's NPV and the way in which this NPV is distributed between rechargers and the basin can shift based on how the administering agency manages and finances the program. The gray bars capture ReNeM's combined NPV before any distributional analysis, with the lighter gray bar showing the reduced NPV when the basin internalizes TPC expenses. The green bars show the basin's distributional NPV under ReNeM when TPC expenses are externally funded, with the light green bar showing the impact incorporation of baseline payments to rechargers has on the basin's NPV. The blue bars similarly show the basin's distributional NPV under ReNeM, but when TPC expenses are internally funded, with the light blue bar again showing the impact incorporation of baseline payments to rechargers has on the basin's NPV. The orange bars show rechargers' distributional NPV, with the light orange bar showing the impact incorporation of a baseline payment has on rechargers' NPV.

5. Future research

Opportunities for further exploration of this study's initial efforts to quantify ReNeM's costs and benefits in the Pajaro Valley include (1) examining the impacts of clustering wet and dry years on infiltration rates, (2) monetizing environmental and social benefits for incorporation into NPV calculations, (3) evaluating projects' recovery efficiency and whether projects' infiltration improves or deteriorates over time, (4) considering project spatial configuration and landscape-level project management impacts recharge, and (5) exploring in more detail the variety of potential regulatory requirements a ReNeM program could encounter and its potential impact on the program's CBA. Notably, some regulatory costs have already been incorporated into this CBA as a component of design and construction. Such regulatory requirements include a Santa Cruz County permit for construction activities and a Stormwater Pollution Prevention Plan. Other potential regulatory costs like water diversion permits and soil disposal permits were not incorporated into this CBA because they have not been necessary in the Pajaro Valley. Though these additional areas warrant exploration, preliminary analysis suggests that adding such details is unlikely to qualitatively alter outcomes.

6. Hydrologic variability and rain gauge data

Hydrologic variability represents an additional element of risk. Because of the structure of NPV calculations using a non-zero discount rate, two time series that have the same total volume of water infiltrated but in different sequences could produce different NPV estimates. Before producing an economic analysis that incorporates hydrologic variability, we first examine the more simplified scenario under which infiltration is modeled to be uniform across all project years at 375 AF/Y across both project sites (SI Fig. 7).

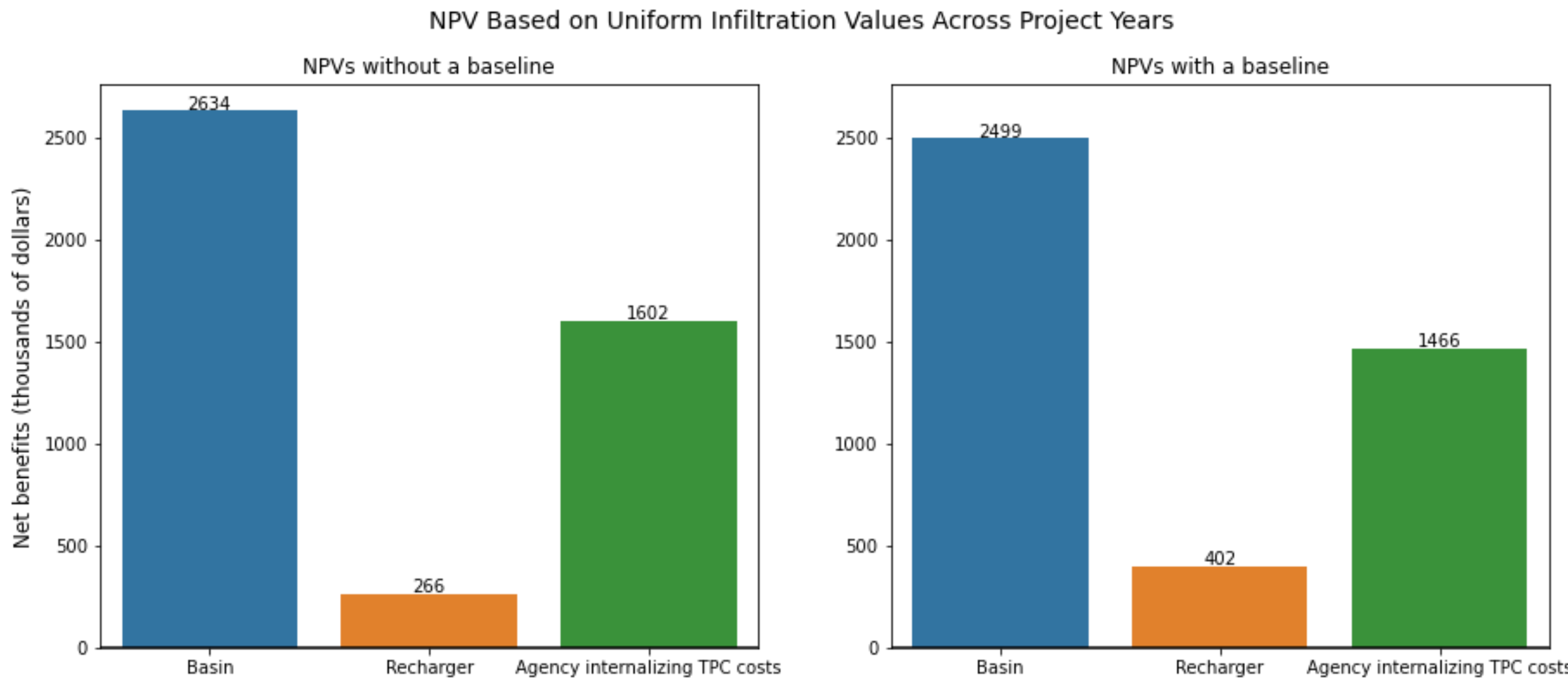

SI Fig. 5: Under a recharge scenario in which ReNeM infiltrates identical volumes of water across all operational years of 375 acre feet per year, the program produces significant net benefits for both the basin (blue) and rechargers (orange), even when the agency acting on behalf of the basin internalizes TPC expenses (green). This remains true, whether or not rechargers receive baseline payments.

A uniform expected infiltration estimate does not reflect hydrologic variability. However, hydrologic variability may affect ReNeM's NPV because NPV is sensitive to the sequence and timing of rebate payments—rebate payments that correspond with infiltration volume and thus respond to hydrologic variability. To model this variability's impact to NPV, we use rain gauge data to generate a variable time series of infiltration data as an input to the NPV equation.

We used monthly rainfall data from the Watsonville Waterworks rain gauge in the Pajaro Valley (data available beginning in 2002 at: https://www.cnrfc.noaa.gov/rainfall_data.php). This rain gauge is closest to PV Water's existing B and KT ReNeM projects, and we assume it is fairly representative of rainfall variability at the B and KT project sites. Using Watsonville Waterworks' monthly data, we calculate rainfall totals per water year (SI Table W) using average monthly rainfall figures where measured data are missing. Using these WY totals, we calculate the average rainfall per year across all available water years. We assume that, had the KT and B ReNeM projects been in operation from WYs 2002 - 2022, they would have infiltrated their average expected yield of 375 AF/Y across both project sites, and use this assumption to construct a linear relationship between rainfall and infiltration across all available water years. We use this linear relationship to generate expected annual infiltration for each WY beginning in 2002. The resulting twenty year time series of variable infiltration data is used in a Monte Carlo simulation to model hydrologic variability's potential influence on NPV calculations.

SI Table 1: Monthly rain gauge data for the Watsonville Waterworks rain gauge from water years 2002 to 2022 indicates that there is significant variability in annual precipitation—variability likely to heavily influence the volume of infiltration ReNeM can produce in any given year. The Watsonville Waterworks rain gauge is located in Watsonville, California, a town in Santa Cruz County in the state's Pajaro Valley.

Water Year	Oct	Nov	Dec	Jan	Feb	Mar	Apr	May	Jun	Jul	Aug	Sep	Water Year Total	Extrapolated infiltration
**2022**	4.12	1.19	9.02	0.17	0.00	1.24	2.22	0.00	0.05	0.06	0.00	1.60	19.67	**353.52**
**2021**	0.00	1.18	1.91	6.15	0.79	2.47	0.11	0.03	0.00	0.07	0.02	0.00	12.73	**228.79**
**2020**	0.00	0.56	10.08	1.93	0.00	3.75	3.01	0.82	0.03	0.00	0.00	0.00	20.18	**362.68**
**2019**	0.10	3.70	2.65	5.23	7.38	5.06	0.38	1.86	0.08	0.01	0.02	0.01	26.48	**475.91**
**2018**	0.05	2.00	M	3.30	0.20	7.25	2.06	0.04	0.00	0.02	0.00	0.00	16.09	**289.18**
**2017**	4.21	2.17	4.86	12.39	9.76	3.18	3.00	0.06	0.08	0.00	0.00	0.13	39.84	**716.02**

**2016**	0.06	3.43	4.99	8.40	1.30	8.03	0.85	0.00	0.01	0.00	0.05	0.00	27.12	**487.41**
**2015**	0.39	3.32	10.14	0.00	2.14	0.66	1.17	0.19	0.00	0.05	0.07	M	19.78	**355.46**
**2014**	0.05	0.46	0.33	0.05	4.74	2.54	1.27	0.05	0.00	0.05	0.01	0.31	9.86	**177.21**
**2013**	0.41	2.98	7.89	0.72	0.53	0.53	0.79	0.00	0.03	0.01	0.00	0.06	13.95	**250.71**
**2012**	1.87	2.10	0.04	2.81	0.65	4.13	3.13	0.01	0.20	0.04	0.00	0.00	14.98	**269.23**
**2011**	1.62	3.23	6.67	2.01	4.65	7.88	0.27	M	1.11	0.01	0.03	0.00	27.58	**495.76**
**2010**	5.31	0.09	3.65	6.12	5.27	2.33	4.19	0.49	0.02	0.01	0.09	0.00	27.57	**495.50**
**2009**	0.35	1.49	3.29	1.15	6.24	2.21	0.36	0.77	0.02	0.02	0.00	0.00	15.90	**285.76**
**2008**	1.26	0.64	2.73	7.37	3.27	0.21	0.28	0.00	0.00	0.00	0.03	0.00	15.79	**283.78**
**2007**	0.10	1.52	4.10	0.96	4.96	0.33	1.06	0.17	0.00	0.00	0.00	0.41	13.61	**244.60**
**2006**	0.17	0.90	8.77	5.51	1.76	8.61	5.81	0.83	0.00	0.00	0.00	0.00	32.36	**581.59**
**2005**	3.51	1.75	4.87	4.22	4.63	5.33	1.59	0.83	0.82	0.03	0.00	0.00	27.58	**495.68**
**2004**	0.41	2.31	7.80	2.49	5.74	0.77	0.40	0.11	0.00	0.00	0.00	0.01	20.04	**360.17**
**2003**	0.00	3.40	8.88	1.27	1.71	1.22	2.52	1.16	0.02	0.02	0.02	0.02	20.24	**363.76**
**2002**	0.34	4.30	7.69	1.48	0.70	1.51	0.24	0.29	0.00	0.00	0.07	0.00	16.82	**302.30**
**Average water year:**													**20.87**	
**ReNeM expected yield divided by average water year:**													**17.97**	

The Monte Carlo simulation generates time series to reflect potential variability in infiltration. The purpose of this exercise is to examine NPV's sensitivity to hydrologic variability, acknowledging explicitly a time-dependent feature of NPV calculations with non-zero rate of pure time preference. To illustrate, a hypothetical 20-year period where all rainfall occurs in the first years would have a higher NPV than a hypothetical 20-year period with the same total infiltration where infiltration occurs at the end of the time series. This variability is an additional element of risk for project proponents. The Monte Carlo simulation selects an infiltration value with replacement from these twenty years of extrapolated infiltration estimates to generate 10,000 simulated time series. These time series illustrate a spread of potential NPV values. SI Fig. 8 captures the overall mean value of this NPV spread as well as the mean value for rechargers and the agency under both a scenario that includes a baseline payment and one that does not.

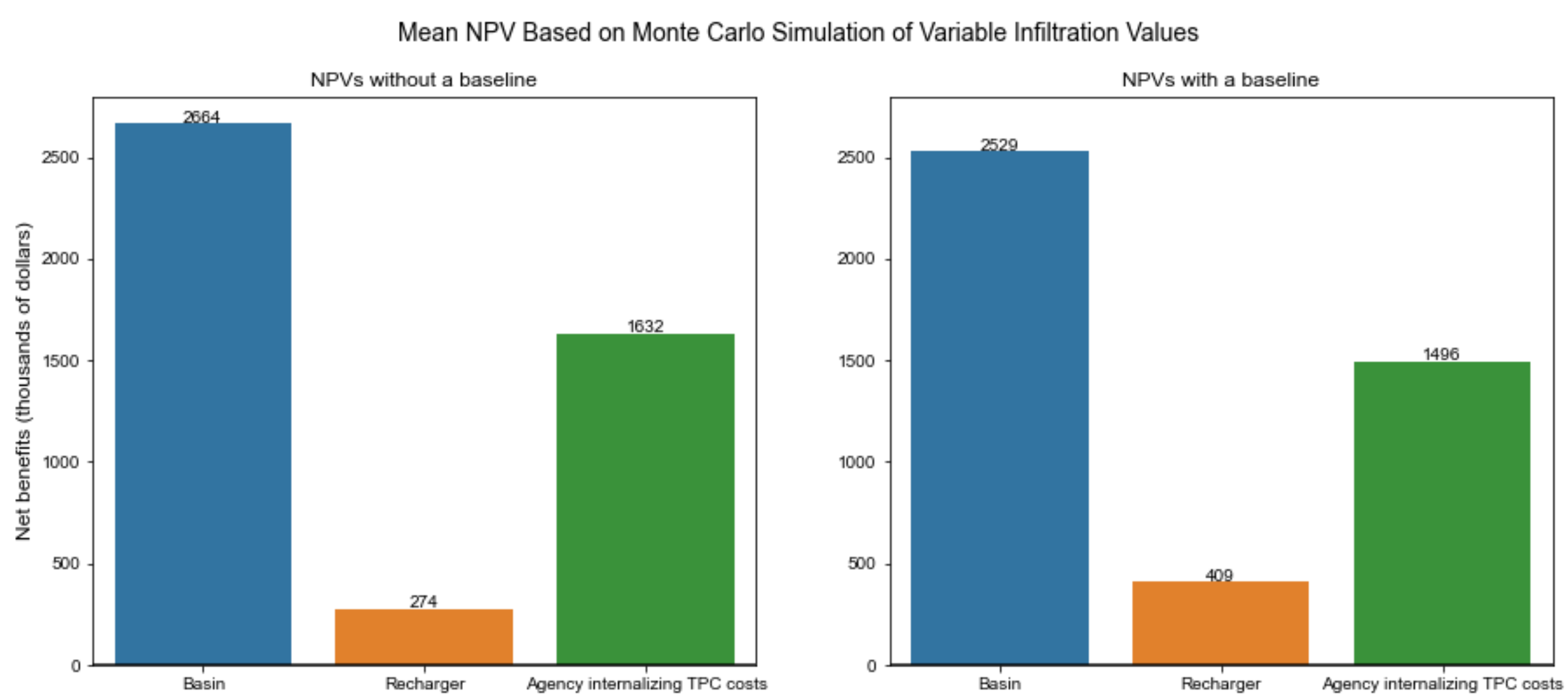

SI Fig. 6: Under a recharge scenario in which ReNeM infiltrates different volumes of water across operational years, the average of which equals 375 acre feet per year, the program produces significant net benefits for both the basin (blue) and rechargers (orange), even when the agency acting on behalf of the basin internalizes TPC expenses (green). This remains true, whether or not rechargers receive baseline payments. Notably, an economic analysis of ReNeM

that incorporates variable infiltration rates produces a slightly higher NPV for both the basin (blue) and rechargers (orange).

Outputs from this methodology produce a ratio between rainfall and infiltration that roughly corresponds with historical data available for the B project site (SI Table X). This value roughly corresponds with the coefficient of 18 derived from rain gauge data:

$$(Q_{Avg}^{B} * rainfall_{Avg}^{B}) * (375/100) = 15$$

SI Table 2: Precipitation and runoff data are available for ReNeM's B project site. Data from water years 2012 to 2022 indicate a relationship between precipitation and runoff that can be coarsely characterized as linear.

Water year	Precipitation (cm)	Precipitation (in)	Runoff collected (m^3)	Runoff collected (AF)	Annual runoff coefficient (—)
**2012**	33.0	13.0	5,590	4.5	0.02
**2013**	39.1	15.4	38,600	31.3	0.14
**2014**	25.6	10.1	41,990	34.0	0.24
**2015**	46.3	18.2	132,150	107.1	0.41
**2016**	69.5	27.4	135,000	109.6	0.28
**2017**	89.7	35.3	175,000	142.1	0.28
**2018**	34.5	13.6	90,995	73.7	0.38
**2019**	55.9	22.0	134,720	109.2	0.35
**2020**	52.0	20.5	68,410	55.4	0.19
**2021**	31.5	12.4	63,320	51.3	0.29
**2022**	41.8	16.4	134,033	108.6	0.46
**Average:**	**47.2**	**18.6**	**92,764**	**75.2**	**0.30**

7. Inflation adjustments

Different values used in this manuscript's calculations were ascertained in different years. For instance, PV Water specifies that its Basin Management Plan, cost estimates for the water management strategies it evaluates are adjusted to 2014 dollars. Meanwhile, some ReNeM project-based cost estimates are more antiquated—items like design and construction costs and the water replacement value—and others are more recent—things like the volumetric pumping fee and management and operation costs. To account for this variation, all values are inflation-adjusted to 2021 dollars. Inflation adjustments were based on Consumer Price Index (CPI) data in SI Table Z and is documented in the manuscript's pertinent code files. The manuscript's main text notes where values have been adjusted for inflation.

SI Table 3: The United States Bureau of Labor Statistics produces Consumer Price Index (CPI) data to track inflation over time—the average change in prices consumers pay for goods and services. This data can be aggregated in different way according to geography, season, sector, and consumer. The CPI data below was aggregated at the monthly timescale to capture inflation statistics for all urban consumers in all US cities, excluding seasonal adjustments. Total annual statistics were then calculated based on available monthly data. The relevant dataset is available at [https://data.bls.gov/timeseries/CUUR0000SA0.](https://data.bls.gov/timeseries/CUUR0000SA0)

Year	Jan	Feb	Mar	Apr	May	Jun	Jul	Aug	Sep	Oct	Nov	Dec	Total
**2010**	216.69	216.74	217.63	218.01	218.18	217.97	218.01	218.31	218.44	218.71	218.80	219.18	**218.06**
**2011**	220.22	221.31	223.47	224.91	225.96	225.72	225.92	226.55	226.89	226.42	226.23	225.67	**224.94**
**2012**	226.67	227.66	229.39	230.09	229.82	229.48	229.10	230.38	231.41	231.32	230.22	229.60	**229.59**
**2013**	230.28	232.17	232.77	232.53	232.95	233.50	233.60	233.88	234.15	233.55	233.07	233.05	**232.96**
**2014**	233.92	234.78	236.29	237.07	237.90	238.34	238.25	237.85	238.03	237.43	236.15	234.81	**236.74**
**2015**	233.71	234.72	236.12	236.60	237.81	238.64	238.65	238.32	237.95	237.84	237.34	236.53	**237.02**

**2016**	236.92	237.11	238.13	239.26	240.23	241.02	240.63	240.85	241.43	241.73	241.35	241.43	**240.01**
**2017**	242.84	243.60	243.80	244.52	244.73	244.96	244.79	245.52	246.82	246.66	246.67	246.52	**245.12**
**2018**	247.87	248.99	249.55	250.55	251.59	251.99	252.01	252.15	252.44	252.89	252.04	251.23	**251.11**
**2019**	251.71	252.78	254.20	255.55	256.09	256.14	256.57	256.56	256.76	257.35	257.21	256.97	**255.66**
**2020**	257.97	258.68	258.12	256.39	256.39	257.80	259.10	259.92	260.28	260.39	260.23	260.47	**258.81**
**2021**	261.58	263.01	264.88	267.05	269.20	271.70	273.00	273.57	274.31	276.59	277.95	278.80	**270.97**
**2022**	281.15	283.72	287.50	289.11	292.30	296.31	296.28	296.17	296.81	298.01	297.71	296.80	**292.66**

8. Other water management projects PV Water has considered

In its BMP, PV Water models the cost and water supply contribution of numerous projects beyond those included in this article's Fig. 1 and Table 3. SI Table Y shows projects that were ultimately omitted from consideration. Notably, many of the projects omitted from further consideration cite cost as a key factor. That this broad range of potential management options are all more expensive further emphasizes ReNeM's cost-competitiveness (see Fig. 1).

SI Table 4: In addition to the projects the Pajaro Valley Water Management Agency's (PV Water) ad-hoc basin management plan (BMP) committee identified as most viable and which the agency plans to undertake, the ad-hoc BMP committee also identified numerous other water management projects ultimately omitted from further consideration for a range of reasons. Notably, many of these projects were omitted from consideration because of high costs.

Water Management Project	Reason for Omission
**Groundwater**	
San Benito County Wells to Aromas Water District (G-1)	Small yield, potential export ordinance issues, compensation requirements
San Benito County Groundwater Demineralization (G-2)	Export ordinance, cost
**Surface Water**	
Imported Central Valley Project (CVP) Water (S-6)	Politically unacceptable
River Conveyance of Mercy Springs CVP Water and Rubber Dam at Murphy Crossing (S-7)	S-11 is more cost effective
Freedom Lake/Corralitos Lagoon (S-8)	Insignificant yield
College Lake Aquifer Storage and Recovery (ASR) in Winter (S-9)	Cost, regulatory uncertainty
Dams at Bolsa and Strawberry Hills with Pajaro Diversion (S-10)	Cost prohibitive
College Lake to Recycled Water Treatment Plant in Summer (S-12)	Less cost-effective than sending water to CDS
Groundwater Recharge Upstream of Murphy Crossing with Water from Soap Lake and San Benito Floodplain (S-13)	Not effective for Pajaro Basin recharge
Partial College Lake to Recycled Water Treatment Plant in Summer (S-14)	Cost-prohibitive
Protection of Natural Recharge Areas and Small-Scale Managed Aquifer Recharge (S-15)	PVWMA is better suited to support others' efforts rather than to manage decentralized program
Zayante Creek Reservoir and Pipeline (S-16)	Cost-prohibitive
Series of Small Dams on Pescadero Creek (S-17)	Insignificant yield
Pipeline from Lexington Reservoir (S-18)	Cost-prohibitive
Warner Lake (S-19)	Insignificant yield
College Lake with Pipeline to Adjacent Farmland (S-20)	S-3 would allow College Lake water to be used at both the coast and inland

Imported Water Supply from Uvas Reservoir (S-21)	Water not available
**Recycled Water**	
Recycled Water to Harkins Slough Recharge Basin (R-1)	Would preclude the use of the recovery wells for S-22
Recycled Water to Harkins Slough and Watsonville Slough Recharge Basins (R-2)	Would preclude the use of the recovery wells for S-2
Pipeline from Santa Cruz WWTP (R-3)	Cost-prohibitive
Pajaro Dunes North Diurnal Recycled Water Storage (R-4)	Cost-prohibitive
Bolsa Dam for Winter Recycled Water Storage (R-5)	Cost-prohibitive
Increased Recycled Water Storage via Grower Ponds (R-7)	Complex to implement with grower/agency coordination
Seasonal Recycled Water Storage South of PVWMA (R-8)	Cost, small yield
Recycled Water from the South County Regional Wastewater Authority (SCRWA) (R-9)	Cost
Winter Recycled Water Advanced Treatment and Injection (R-10)	Cost
Dams at Bolsa and Strawberry Hills for Recycled Water Storage (R-12)	Cost, small yield
**Demand Management**	
Increased Irrigation Efficiency with Soil Tensiometers (D-1)*	Combined with D-4 & -5 to create D-7
Fallow 10% of Farmland (D-2)	Politically & economically unacceptable
Fallow 8,000 Acres of Coastal Land (D-3)	Politically & economically unacceptable
Irrigation Efficiency Training (D-4)*	Combined with D-1 & -5 to create D-7
Performance-based Water Conservation Incentives (D-5)*	Combined with D-1 & -4 to create D-7

1
