# Supplementary material for: Recharge net metering (ReNeM) is a novel, cost-effective management strategy to incentivize groundwater recharge
Source: Nat Water. 2023 Oct 18;1(10):855–63. doi: 10.1038/s44221-023-00141-1 (PMC13533828; doi:10.1038/s44221-023-00141-1)
Supplement: Supplementary file 2 [file 44221_2023_141_MOESM2_ESM.pdf]

nature portfolio

Corresponding author(s): Michael Kiparsky, Molly Bruce

Last updated by author(s): Sep 6, 2023

Reporting Summary

Nature Portfolio wishes to improve the reproducibility of the work that we publish. This form provides structure for consistency and transparency in reporting. For further information on Nature Portfolio policies, see our Editorial Policies and the Editorial Policy Checklist.

Statistics

For all statistical analyses, confirm that the following items are present in the figure legend, table legend, main text, or Methods section.

n/a	Confirmed	
☐	☒	The exact sample size (n) for each experimental group/condition, given as a discrete number and unit of measurement
☐	☒	A statement on whether measurements were taken from distinct samples or whether the same sample was measured repeatedly
☐	☒	The statistical test(s) used AND whether they are one- or two-sided<br>*Only common tests should be described solely by name; describe more complex techniques in the Methods section.*
☒	☐	A description of all covariates tested
☐	☒	A description of any assumptions or corrections, such as tests of normality and adjustment for multiple comparisons
☐	☒	A full description of the statistical parameters including central tendency (e.g. means) or other basic estimates (e.g. regression coefficient) AND variation (e.g. standard deviation) or associated estimates of uncertainty (e.g. confidence intervals)
☒	☐	For null hypothesis testing, the test statistic (e.g. F, t, r) with confidence intervals, effect sizes, degrees of freedom and P value noted<br>*Give P values as exact values whenever suitable.*
☐	☒	For Bayesian analysis, information on the choice of priors and Markov chain Monte Carlo settings
☒	☐	For hierarchical and complex designs, identification of the appropriate level for tests and full reporting of outcomes
☒	☐	Estimates of effect sizes (e.g. Cohen's d, Pearson's r), indicating how they were calculated

Our web collection on statistics for biologists contains articles on many of the points above.

Software and code

Policy information about availability of computer code

Data collection	Custom code was developed for this research to pull in data files in order to complete both analyses of inputs and communication of results. Code was generated in Python using Jupyter Notebooks and is available in distinct GitHub repositories under the root hyperlink, https://github.com/mollymbruce.
Data analysis	Custom code was developed for this research to pull in data files in order to complete both analyses of inputs and communication of results. Code was generated in Python using Jupyter Notebooks and is available in distinct GitHub repositories under the root hyperlink, https://github.com/mollymbruce.

For manuscripts utilizing custom algorithms or software that are central to the research but not yet described in published literature, software must be made available to editors and reviewers. We strongly encourage code deposition in a community repository (e.g. GitHub). See the Nature Portfolio guidelines for submitting code & software for further information.

Data

Policy information about availability of data

All manuscripts must include a data availability statement. This statement should provide the following information, where applicable:

- Accession codes, unique identifiers, or web links for publicly available datasets
- A description of any restrictions on data availability
- For clinical datasets or third party data, please ensure that the statement adheres to our policy

All data generated by this study or analyzed as part of this research are included in this published article and its supplementary information files.

Human research participants

Policy information about studies involving human research participants and Sex and Gender in Research.

Reporting on sex and gender	Not applicable
Population characteristics	Not applicable
Recruitment	Not applicable
Ethics oversight	Not applicable

Note that full information on the approval of the study protocol must also be provided in the manuscript.

Field-specific reporting

Please select the one below that is the best fit for your research. If you are not sure, read the appropriate sections before making your selection.

☐ Life sciences ☒ Behavioural & social sciences ☐ Ecological, evolutionary & environmental sciences

For a reference copy of the document with all sections, see nature.com/documents/nr-reporting-summary-flat.pdf

Behavioural & social sciences study design

All studies must disclose on these points even when the disclosure is negative.

Study description	This economics assessment used quantitative methods to understand the costs and benefits of the Recharge Net Metering (ReNeM) program and to numerically compare the program to other water management options.
Research sample	This study incorporated data from all existing ReNeM projects that had a threshold longevity of operation to ascertain data on both upstart costs and continued operation & maintenance costs, particularly under varied hydrological conditions. Given ReNeM's status as a fairly young water management strategy, this sample size was necessarily small, though it is representative of all data available for multi-year program operation.
Sampling strategy	Because the research sample size was small, all available data relevant to this economics assessment from the above-described ReNeM project sites were utilized for this assessment.
Data collection	Data were collected using a range of methods. Hydrological data was collected using in-situ monitoring equipment including stream gage monitoring equipment, flow meters, and water quality monitoring equipment. Collected data were captured by computer. Financial data was collected from receipts, contracts, reports, and personal accounts. All data collection predate this economics assessment and those involved in data collection were unaware of a future assessment or the likely implications their data would have on that assessment.
Timing	Data collection captured in this economics assessment began in 2002 and extends through present.
Data exclusions	No data from the research sample were excluded from analysis.
Non-participation	There were no instances of non-participation or attrition.
Randomization	Data participants were not separated into experimental groups.

Reporting for specific materials, systems and methods

We require information from authors about some types of materials, experimental systems and methods used in many studies. Here, indicate whether each material, system or method listed is relevant to your study. If you are not sure if a list item applies to your research, read the appropriate section before selecting a response.

Materials & experimental systems

n/a	Involved in the study
☒	☐ Antibodies
☒	☐ Eukaryotic cell lines
☒	☐ Palaeontology and archaeology
☒	☐ Animals and other organisms
☒	☐ Clinical data
☒	☐ Dual use research of concern

Methods

n/a	Involved in the study
☒	☐ ChIP-seq
☒	☐ Flow cytometry
☒	☐ MRI-based neuroimaging
